# Improving Protein Subcellular Location Classification by Incorporating Three-Dimensional Structure Information

**DOI:** 10.3390/biom11111607

**Published:** 2021-10-29

**Authors:** Ge Wang, Yu-Jia Zhai, Zhen-Zhen Xue, Ying-Ying Xu

**Affiliations:** 1 School of Biomedical Engineering, Southern Medical University, Guangzhou 510515, China; wg19960730@163.com (G.W.); zz.xue@siat.ac.cn (Z.-Z.X.); 2 Guangdong Provincial Key Laboratory of Medical Imaging Processing, Southern Medical University, Guangzhou 510515, China; 3 Guangdong Province Engineering Laboratory for Medical Imaging and Diagnostic Technology, Southern Medical University, Guangzhou 510515, China; 4 Guangzhou Women and Children’s Medical Center, Department of Pharmacy, Guangzhou Medical University, Guangzhou 510623, China; kapono@126.com; 5 Paul C. Lauterbur Research Center for Biomedical Imaging, Shenzhen Institutes of Advanced Technology, Chinese Academy of Sciences, Shenzhen 518055, China

**Keywords:** subcellular location prediction, protein structure, deep learning, graph neural network, protein data bank

## Abstract

The subcellular locations of proteins are closely related to their functions. In the past few decades, the application of machine learning algorithms to predict protein subcellular locations has been an important topic in proteomics. However, most studies in this field used only amino acid sequences as the data source. Only a few works focused on other protein data types. For example, three-dimensional structures, which contain far more functional protein information than sequences, remain to be explored. In this work, we extracted various handcrafted features to describe the protein structures from physical, chemical, and topological aspects, as well as the learned features obtained by deep neural networks. We then used these features to classify the protein subcellular locations. Our experimental results demonstrated that some of these structural features have a certain effect on the protein location classification, and can help improve the performance of sequence-based location predictors. Our method provides a new view for the analysis of protein spatial distribution, and is anticipated to be used in revealing the relationships between protein structures and functions.

## 1. Introduction

Given that subcellular/organelle structures in cells provide specific physiological and functional environments, the determination of the subcellular locations of proteins is believed to be an important aspect of the understanding of their functions [1,2]. In the past decades, automated bioinformatics algorithms have gained popularity in location determination tasks.

Most of the methods that were developed for the prediction of protein subcellular localizations employed amino acid sequences as the information source because the protein sequence data are abundant and easy to access [3]. The theoretical basis of the predictions is that one protein is transported into specific subcellular structure(s) according to its signal peptide, which is a short segment buried in the amino acid sequence. Some prediction methods, such as Hum-mPLoc 3.0 [4] and SCLpred [5], constructed sequence features through a target signal search, motif analysis, or homology transfer, while some works in recent years, like DeepLoc [6] and HumDLoc [7], employed deep learning models to learn the protein features automatically.

Three-dimensional (3D) protein structures, as a complex representation of proteins, contain spatial folding information (such as secondary structure elements, topological domains, and the protein surface) that largely manipulate biological functions, such as binding specificity and signal transduction. Given that the 3D structures stored in the protein data bank (PDB) database [8,9] are increasingly intensive, some studies have begun to use these 3D structures as source data to predict protein topological domains [10,11,12], molecule interpretation [13], the amino acid environment [14], enzyme types [15,16], and protein functions [17,18].

Signal patches in the tertiary structures can be indicators for subcellular location [19,20,21]. A signal patch is a specific 3D arrangement on the surface of a folded protein, and can be discontiguous, in contrast to signal peptides. To date, few works have attempted to incorporate protein structure information into a location analysis. The description of structures include the amino acid composition of secondary structures (α-helix, β-strand, and loop) [22,23], the amino acid composition of surface residues [24], and the average chemical shift related to secondary structures [25]. These features can aid location classification models, but all of them are manually defined and only focus on single aspects, which are too simple to sufficiently grasp global structural information.

In this paper, we seek to construct a comprehensive representation for 3D protein structures, which can perform well in the classification of protein subcellular locations. These features are designed or have learned to describe information underlying the 3D structures in terms of the topological structures, arrangement, and physicochemical properties of atoms and other organelle-related characteristics. According to our experimental results, some of the structural features were demonstrated to be effective in protein location prediction, and can increase the classification performance when combined with sequence features. This work provides a new perspective on protein subcellular location research.

## 2. Materials and Methods

### 2.1. Dataset

In this study, we constructed a new protein structure benchmark dataset from the PDB database (https://www.rcsb.org, accessed on 28 October 2021), which stores hundreds of thousands of biological macromolecules obtained through experimental methods, such as X-ray crystallography, nuclear magnetic resonance spectroscopy, and electron microscopy. The dataset was generated by applying PISCES (Dunbrack Lab, Philadelphia, PA, USA) [26] with an identity cutoff of 25% to a previous dataset [27] in order to remove redundant protein sequences. The final benchmark dataset has 1052 proteins. Each of the proteins has a PDB file, containing the primary and secondary structures of the protein, an atom list and the residues they belong to, and atomic 3D coordinates. It is noted that most of the protein structures in the PDB are fragments that do not cover the entire amino acid chains. In our dataset, about 15% of the proteins have complete structures (covering over 99% of the amino acid chain). Detailed information is shown in Supplementary Table S1.

These proteins localize specifically to one of four major subcellular locations, namely the nucleoplasm, plasma membrane, cytosol, and mitochondria. The subcellular locations were derived from the Swiss-Prot database (https://www.uniprot.org, accessed on 28 October 2021), a widely used protein sequence database [28]. In order to ensure the quality of the data, we only considered the subcellular locations labeled with ECO: 0000269, which indicates that the locations are supported by experimental evidence. The number of proteins in each subcellular location is shown in Table 1.

### 2.2. Protein Structural Features

A robust feature description of 3D protein structures is important for effective protein classification. In this paper, we tested eight handcrafted and learned descriptors for proteins, and all of them can ensure invariance to structure rotation. Table 2 summarizes the descriptors, which were divided into four categories, namely physical features, chemical features, topological features, and deep learning features. The features are described below, individually.

#### 2.2.1. Physical Features

The spatial position of each amino acid in the protein chain determines the protein’s 3D shape [29]. Here, we understood the shape of the proteins at the atomic level and extracted the relevant features. We used three physical descriptors, namely the contact matrix, dihedral angle features, and sub-structure frequency features.

##### Contact Matrix

**Figure 1:**
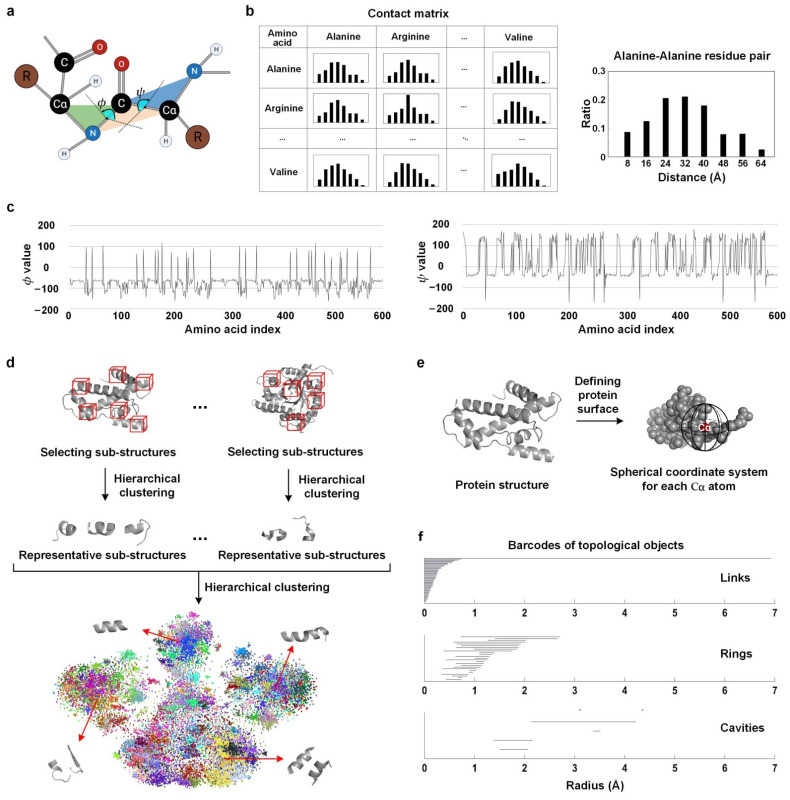
Illustration of the structural protein features. (**a**) Definition of the Cα atom and dihedral angles *φ* and *ψ*. A pair of amino acids are shown, and the two brown circles represent side-chain functional groups. (**b**) Distance matrix calculated for the protein C1-THF synthase (PDB id: 1a4i). The enlarged bar chart of alanine–alanine is given. (**c**) Curves of dihedral angles. The horizontal axis represents the chain of protein 1a4i. (**d**) Process of detecting globally representative sub-structures for frequency feature extraction. Sub-structures were selected and used in a two-step clustering step to obtain representative patterns. Here, the sub-structures of all of the proteins in our dataset were visualized by t-SNE. Different colors represent different clusters, and some of the most representative sub-structures were shown. (**e**) Definition of the surface amino acids. If an amino acid has neighbors at all of its 12 spatial areas, it is not a surface amino acid; otherwise, it is. (**f**) Barcodes of the persistent homology of protein 1a4i.

For each protein, we constructed a contact matrix to describe the spatial distances among the amino acids [15]. In order to calculate the precise distances, each amino acid was represented by its unique Cα atom, which is a carbon atom connected to the functional group in the amino acid backbone (Figure 1a). All of the distance values were measured among the Cα atoms. For each amino acid pair, the distribution of the distances was divided into eight bins ranging from 0 to 64 Å (Figure 1b). Thus, we constructed an 8 × 20 × 20 contact matrix for each protein. Considering that the distance matrix is symmetrical, we removed the repeated elements, and the dimensionality became 1680 (8 × 210). In order to reduce feature redundancy, we used stepwise discriminant analysis [30] to select the most informative features, and this led to a 216–256 dimensional feature vector for each protein (which varies in 10-fold cross validation).

##### Dihedral Angle Features

Dihedral angles describe the deflection of adjacent amino acids in the protein backbone. The dihedral angles are highly correlated with the secondary structures of proteins, providing important information about local tertiary structures [31]. Each amino acid link has two dihedral angles, namely *φ* and *ψ* (Figure 1a). *φ* is determined by Cα-N-C-Cα, and is the angle between the Cα-N-C plane and the N-C-Cα plane. *ψ* is determined by N-C-Cα-N, and is the angle between the N-C-Cα plane and the C-Cα-N plane. The two angles used the N-C-Cα plane as a base, and the values of the two dihedral angles are between −180° and 180°. We firstly drew two curves of dihedral angles for each protein chain (Figure 1c) and then extracted 15 frequency domain and wavelet transform features (Supplementary Table S2) from each curve as the protein features.

##### Sub-Structure Frequency

Some specific sub-structure components of the 3D structures dominate the protein functions [32], so we proposed to use the frequencies of compact sub-structures to represent the protein (Figure 1d). First, we detected compact sub-structures based on the distance matrices. The size of each distance matrix was the length of the protein chain, and the elements were the spatial distances of the corresponding amino acid pairs. Each matrix was walked through by a sub-matrix window of size 10 × 10, with a step size of 5. The sub-structures that have an average distance less than 15 Å were selected as tightly connected structures. Some selected sub-structures were composed of discontiguous amino acids.

After extracting the compact sub-structures of all of the proteins, we tried to cluster the sub-structures. Each sub-structure was represented by the 100-dimensional expanded distance matrix elements and 20-dimensional amino acid composition (AAC) features. The total number of sub-structures was over one million, and the large number of sub-structures hindered the implementation of direct clustering, so we used a two-step clustering method. In the first step, hierarchical clustering was performed on each protein individually, and the clustering centroids of the sub-structures for each protein were obtained. The local clustering centroids were believed to be representative of the source proteins. The second step performed a hierarchical clustering on only the local clustering centroids of all of the proteins, and obtained the global clustering centroids. The distance thresholds of the two hierarchical clusterings were determined by a grid search.

Finally, the sub-structure frequency features were constructed by assigning each sub-structure to the closest cluster centroid and counting the proportion of sub-structures of one protein across the clusters.

#### 2.2.2. Chemical Features

The chemical properties of the surfaces of protein molecules determine the interaction between the proteins and the outside world [33]. As such, this study attempted to use the composition and chemical properties of the protein surface to represent the proteins.

The surface amino acids of a protein are defined by measuring whether the amino acid is fully surrounded. We took the Cα atom of each amino acid as the center of gravity, and constructed a spherical coordinate system (Figure 1e). The spherical coordinate system was then divided into 12 spatial areas equally. If at least one area does not have any neighbor Cα atoms, then the center amino acid was regarded as a surface one.

The 20-dimensional AAC features of the surface amino acids were calculated. In addition, according to the chemical properties of the amino acid [17] (Supplementary Table S3), we calculated 22 chemical features, such as chargeability, polarity, hydrophobicity, and hydrophilicity. For each protein, the chemical features were defined by the expectations of amino acid properties:

(1) $$f^{\left(i\right)}=\sum_{j=1}^{n}\left(\alpha_{j}\times p_{j}^{\left(i\right)}\right)$$

where *f*^(*i*)^ represents the *i*-th chemical feature, *n* is the number of amino acids (*n* = 20), *α_j_* is the proportion of the *j*-th amino acid, and $p_{j}^{\left(i\right)}$ is the value of the *i*-th chemical property of the *j*-th amino acid in Supplementary Table S3.

#### 2.2.3. Topological Features

The topology of a protein structure is a simplified description of its fold, including the amino acid nodes and their relative positions and links [34]. Here, we constructed the protein features by analyzing the 3D structures in terms of the positional relationship of the topological structures.

##### Gauss Integrals

The Gauss integrals’ features were obtained using Gauss integrals (SourceForge, San Diego, CA, USA) to calculate the global writhe of the space curve [35]. We used it to describe the degree of twisting of the 3D structures of the proteins. The protein backbones were represented as space curves connecting the Cα atoms. A set of defined quantitative features describing crossings in the planar projections of the protein curves were extracted [36]. The dimensionality of the Gauss integral descriptor was 31.

##### Persistent Homology

Persistent homology features [37] show the persistence of topological components in different scale spaces. Persistent homology involves two concepts, namely the homology group and connectivity number [38]. The process of calculating persistent homology features began by regarding each amino acid as a 3D ball centered at the Cα atom. When increasing the radii of these balls, the boundary of one ball would include the center of another Cα, and the two amino acids would be considered as being linked. With increasing radii, topological objects—such as links, rings, and cavities—continuously appear over a range of spatial scales. The appearance and disappearance of these topological objects were recorded as barcodes. An example of the barcodes of one protein is shown in Figure 1f, where each barcode panel represents a specific topology type. In this process, the homology group and connectivity number of the amino acids would be changed. We used 34-dimensional features—including the number of the barcodes, the number of starting and ending points of the barcodes in a certain radius range, the minimum starting points, and the maximum ending points—to describe the barcodes and represent the proteins.

#### 2.2.4. Deep Learning Features

Compared with the manually designed features, deep learning methods can automatically extract intrinsic information from the 3D protein structures. Here, we used deep convolutional neural networks (CNNs) and graph convolutional neural networks (GCNs) to learn the structural features.

##### CNN Features

Given that some CNN architectures have been demonstrated to be effective in extracting protein structure information, we used transfer learning based on a previous CNN network of protein function classification [15]. The input of the network was a set of two-dimensional feature maps constructed by the contact distances between the amino acids and dihedral angles. The network architecture employed three computational blocks of consecutive convolutional, batch normalization, rectified linear unit activation, dropout and max-pooling layers, and a fully connected layer. Based on the pre-trained network, we replaced the output layer, froze the parameters of the previous layers, and used our training data to fine-tune the parameters of the last two layers. The Softmax loss function was employed, and stochastic gradient descent was used for the parameter optimization. After the fine-tuning, we used the feature map of the penultimate layer as the protein features. The dimension of the extracted CNN features was 1000, and was reduced to 108~151 (which varies in 10-fold cross validation) by using stepwise discriminant analysis.

##### GCN Features

**Figure 2:**
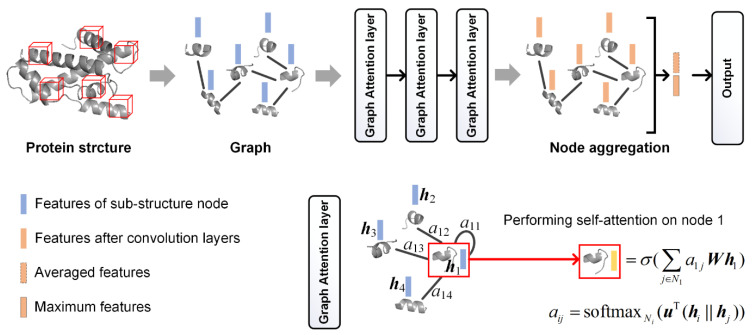
Graph convolutional neural network used for the learning of the protein representations. The graph attention layers were illustrated, where ***h****_i_* represents the feature vector of node *i*, *a_ij_* represents the attention coefficient indicating the importance of node *j* to node *i*, *N_i_* represents the neighborhood of node *i*, (***h****_i_* || ***h****_j_*) represents the concatenation of ***h****_i_* and ***h****_j_*, and ***u*** and ***W*** represent the weights to transform features into higher-level features, and would be optimized in the training phase. The function softmax of *N_i_* represents the normalizing of the attention coefficients across all of the neighbors of node *i*, and σ(.) is a nonlinearity function (here, we used ReLU).

One alternative way to show the 3D structure of a protein is a graph; as such, we attempted to use the GCN model [39] to create protein representations (Figure 2). In order to convert each protein into a graph, we used compact sub-structures as nodes, and considered the close sub-structures as connected. The selection of the sub-structures used the same method as that in the sub-structure frequency feature extraction. The only difference was that the threshold of the average inner distance for the screening of compact sub-structures was set as 10 Å to ensure sufficient sub-structures. Two sub-structures were connected with an edge if the distance between them was less than 10 Å, where each centroid was the average coordinate of all of the Cα atoms in one sub-structure. On average, one protein graph has 118 nodes and 1907 edges.

The architecture of the graph neural network is illustrated in Figure 2. In order to construct the network inputs, each sub-structure node was encoded by the 20-dimensional AAC features and 22-dimensional chemical property features, as described previously. The network has three graph attention layers in series [40]. These layers enable the calculation of different weights for different nodes in a neighborhood in the graph convolution, and have been demonstrated to be effective in graph classification [41,42]. In order to aggregate all of the nodes in one graph, the features were then averaged across all of the nodes, and the maximum of each dimension of the features was retained and concatenated with the average feature vector. The following was the output classification layer. The weights in the network were optimized using the ADAM method, based on the training data. After the optimization, 84 features from the aggregation layer were extracted as the protein features.

## 3. Results and Discussion

### 3.1. Classification Results of Single Descriptors

**Figure 3:**
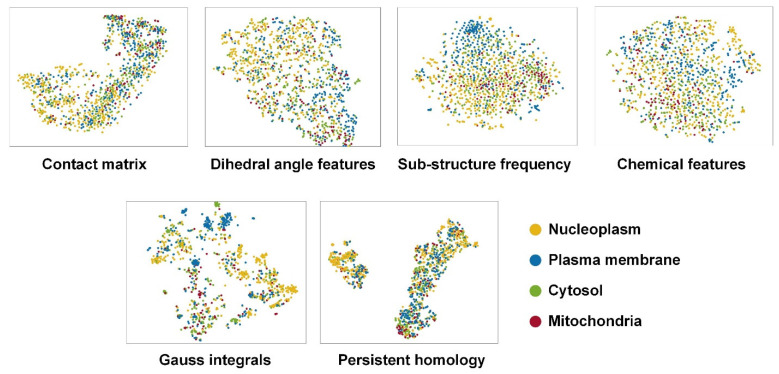
t-SNE visualization of the structural features.

In order to test the ability of the above descriptors to distinguish protein subcellular locations, we used t-distributed stochastic neighbor embedding (t-SNE) to visualize the dimension reduction projection of the handcrafted descriptors (Figure 3). The cytosol and mitochondria were severely mixed, and some descriptors such as sub-structure frequency and Gauss integrals can discriminate the plasma membrane and nucleoplasm relatively well. The visualization results indicated that the protein structure information, to some extent, can distinguish different protein subcellular location patterns.

In addition, we employed a support vector machine (SVM) and random forest (RF) to build protein location classifiers for these handcrafted descriptors. Libsvm-3.24 was used to build the SVM models [43]. The model parameters including *g* and *c* in the SVMs, and number of trees in the RFs was determined by a grid search. A 10-fold cross validation was employed, in which the protein dataset was randomly partitioned into 10 equally sized subsets. Of the 10 subsets, a single subset was retained as the validation set, and the remaining nine subsets were used as training data. The process was repeated 10 times, with each of the subsets being used once as the validation set. We used the accuracies and F1 scores obtained by the 10-fold cross validation to evaluate the model performance.

**Figure 4:**
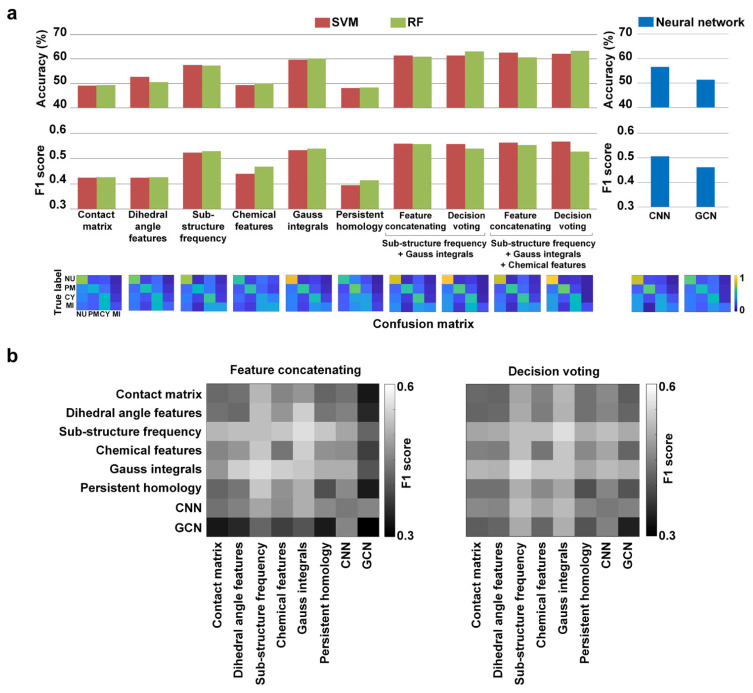
Protein subcellular location classification results of the structure features. (**a**) Results of single descriptors, two-feature and three-feature combinations. The confusion matrices were calculated based on results from the RF models and network models. NU: nucleoplasm; PM: plasma membrane; CY: cytosol; MI: mitochondria. ‘+’ represents the fusion of two feature sets. (**b**). The F1 scores obtained by the SVM models of the fusing descriptors.

Figure 4a shows the classification results of the handcrafted features, as well as the deep neural network results. On one hand, the RFs slightly outperformed the SVM models, and all of the features of the four aspects showed some discriminative ability. The Gauss integral descriptor with the RF achieved the best performance, with an accuracy of 60.16% and an F1 score of 0.54. Meanwhile, the designed sub-structure frequency features obtained a promising result. Both of the Gauss integrals and the sub-structure frequency features attempted to describe structural components, indicating that this aspect could be effective in protein subcellular location classification. On the other hand, the deep neural networks did not achieve the expected high performance. One possible reason is that the amount of training data was limited, which suggests that we need to collect a large number of protein structures for training in future works.

### 3.2. Results of Fusing Different Descriptors

In order to build an efficient feature set of protein structures for location analysis, we performed feature fusing and tested whether multiple descriptors can yield better results. Two fusion methods, namely feature concatenating and decision voting, were used. The first method concatenated the feature vectors after the feature selection, and then fed them into classification models, while the second method averaged the output scores from the models trained on individual descriptors. Here, the derived CNN and GCN features were also used in the feature fusion. The heat maps in Figure 4b show the F1 scores obtained by combining each two of the descriptors. The decision voting led to higher performance than the feature concatenating overall. For the combination performance, the best combination is sub-structure frequency combined with Gauss integrals’ features, and the comparison results are shown in Figure 4a.

Furthermore, we tested the combinations of three or four descriptors. The combination of the sub-structure frequency, Gauss integrals, and chemical features was the best-performing, and can achieve improvements of 3.27% accuracy and 0.028 F1 score compared to the best single descriptor (Figure 4a). The confusion matrices shown indicate that the combined models could enhance the prediction and alleviate the impact of data imbalance. The combinations of more than three descriptors cannot achieve better performance, which is likely because of information redundancy. Thus, the constructed optimal feature set for the protein structure would contain three descriptors, namely the sub-structure frequency, Gauss integrals, and chemical features. The features describe the protein structures from different views, such that their heterogeneity and complementarity help to enhance the accuracy of the combined models.

### 3.3. Improvements by the Incorporation of Sequence Features

We also investigated the improvements achieved when incorporating the structure information with protein sequence features. Here, two sequence-based predictors of protein subcellular location, namely, Hum-mPLoc 3.0 [4] and DeepLoc [6], were utilized. The former proposed a multi-view feature set, including residue statistical features, gene ontology annotation correlation features, and peptide-based functional domain features, while the latter derived features from a well-trained deep learning model that utilized a recurrent neural network to process the sequences. We combined the optimal structure feature set (containing the sub-structure frequency, Gauss integrals, and chemical features) with these sequence-based models at the feature and decision levels, respectively. The feature-level fusion employed multiple kernels for the SVMs [44] and performed feature concatenating for the RF models, while the decision level used weighted fusion according to the model performance [45]. Compared with feature concatenating, the multi-kernel learning allowed us to adjust the weighting between the sequence and structure features, and can achieve a higher incorporation performance (Supplementary Figure S1).

**Figure 5:**
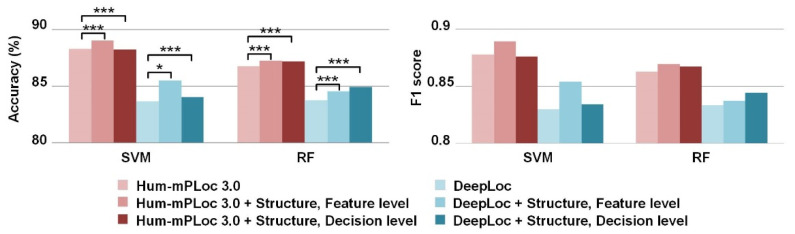
Results of classification models before and after incorporating the protein structure features. The differences between the groups are marked with * and ***, corresponding to the significance levels of 0.05 and 0.001, respectively.

The results of the 10-fold cross validation are presented in Figure 5. Three observations can be found. First, incorporating the structure features achieved a better performance than the sequence-based models. A paired *t*-test was conducted on the output probability scores to measure whether the differences between the models before and after incorporating the structure features are significant. Most of the cases obtained a *p*-value of <0.001. This finding demonstrated that feature extraction from 3D protein structures could capture a large amount of information that cannot be learned from sequences. Second, the fusion at the feature level achieved a better performance than that at the decision level, as the multi-kernel mechanism can work well in feature fusion. Third, the kernel weights which searched for Hum-mPLoc 3.0 and the structure features were 0.83 and 0.17, respectively, while those for DeepLoc and structure features were 0.57 and 0.43, respectively. This finding indicated that the two parts of the information played important roles in the protein location classification.

**Figure 6:**
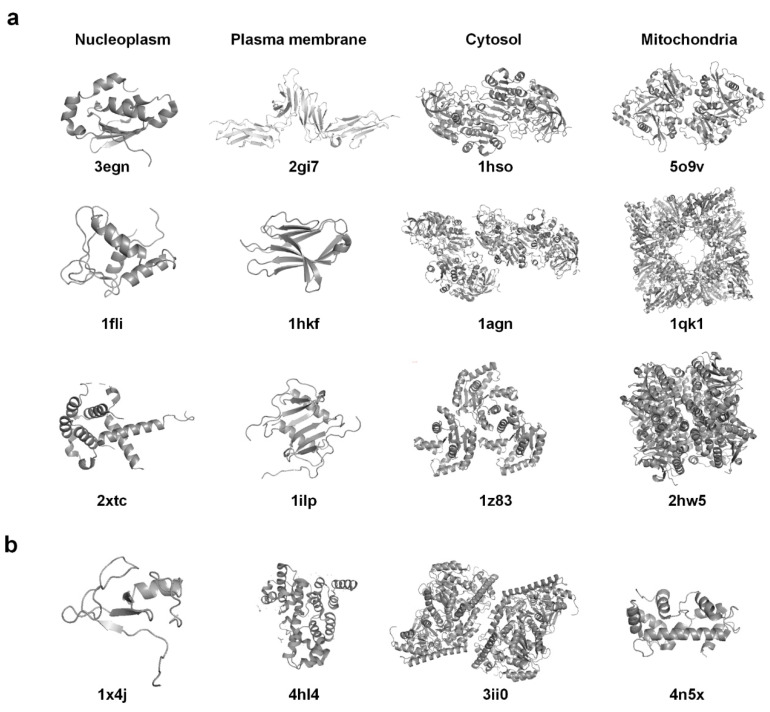
Protein structure examples of different subcellular locations. (**a**) The top three structures with the highest predicted probabilities for each subcellular locations are listed. The predicted probabilities are from the model that combined Hum-mPLoc 3.0 and the structure information at the feature level. (**b**) Structure examples that were wrongly predicted by sequence based predictors but received correct predictions from the incorporated models.

In order to obtain an intuitional observation of the predictions, we also listed some specific protein structures in Figure 6. For each of the four subcellular locations, the top three structures that had the highest prediction scores from the best-performing combined model are shown. It can be seen that the listed plasma membrane structures are related to soluble fragments of the proteins, rather than the fragments embedded in membranes like transmembrane regions. The reason was that only 20 membrane proteins in our dataset have structures covering the transmembrane regions, and the classification models are likely to be manipulated by the features of the non-membrane regions. The structures in our dataset were released in PDB between 1992 and 2020, and most of them were resolved by X-ray diffraction or nuclear magnetic resonance. It is challenging for these methods to resolve membrane proteins because of their large size and poor crystallization. Although the use of cryo-electron tomography (cryo-EM) increased the number of membrane protein structures in recent years, the proportion was still relatively small. In our dataset, there are only nine proteins, of which five are membrane proteins, as solved by the cryo-EM method. It is anticipated that more complete structures of membrane proteins could be resolved in the future, and the characteristics which differentiate them from non-membrane proteins could be extracted and used in the classification. In addition, the protein examples of cytosol and mitochondria seemed quite similar in their structures, which might be the reason why the two classes were indistinguishable in our experiments. We also provided one example structure for each subcellular location in Figure 6b, where the prediction classes of these example structures were corrected after incorporating the structure features, showing that the structure information played quite an effective role in their location classification.

## 4. Conclusions

In this work, we constructed a multi-view representation for 3D protein structures and demonstrated its utility for protein subcellular location analysis. This work has two main contributions. First, this study built a structure representation from a wide variety of perspectives, which facilitated the search of location-related information in the protein structures. Some approaches, including the calculation of sub-structure frequency and the GCN framework, were designed and first used in profiling the protein localization. Our experimental results suggest that some methods can achieve promising results. Second, this study demonstrated that incorporating 3D structure information can improve the performance of protein subcellular localization classification. Hence, protein structures might contain some location-related information (like signal patches) that do not exist in amino acid sequences.

Currently, the discovery of the relationship between 3D protein structures and subcellular locations is still in its infancy stage. Our future work will search for more effective structure descriptors and further explore the use of deep neural networks in this field. Although the performance of the current neural networks is not good, the models are likely to benefit from more structure data in future works. The predictions of the entire human proteome by AlphaFold, which is a state-of-the-art machine learning model for protein structure prediction, have been made freely and openly available very recently [46], giving an abundant data source for the improvement of our network models. Other efforts to enhance the network performance can be made in terms of graph construction, node representation, network architecture, and more skills for model training. The attention mechanism and gradient-weighted class activation map in the network model can help detect dominant and informative sub-structures for the elaboration of their functions in protein localization in the future.

## Figures and Tables

**Table 1:** Number of proteins in our dataset.

Subcellular Location	Number of Proteins
Nucleoplasm	385
Plasma membrane	281
Cytosol	275
Mitochondria	111
Total	1052

**Table 2:** Summary of the feature types.

Feature Category	Descriptor	Brief Description	Feature Dimension
Physical features	Contact matrix	Statistics of distances between amino acid pairs	1680
	Dihedral angle features	Frequency domain and wavelet transform features extracted from dihedral angle curves along protein chain	30
	Sub-structure frequency	Frequencies of small sub-structures of one protein in clusters	184
Chemical features	Chemical features	Composition and chemical properties of surface amino acids	42
Topological features	Gauss integrals	Type and number of crossings in planar projections of protein curve	31
	Persistent homology	Features extracted from persistent barcodes of topological objects in protein structure	34
Deep learning features	Convolutional neural network features	Features learned by a convolutional neural network with contact distances and dihedral angles as input	1000
	Graph convolutional neural network features	Features learned by a graph convolutional network with attention layers	84

## Data Availability

The dataset and code used in this study are available at https://github.com/PRBioimages/Protein-structure-features (accessed on 28 October 2021).

## Supplementary Materials

The following are available online at https://www.mdpi.com/article/10.3390/biom11111607/s1. Figure S1: Comparison of the incorporating methods at the feature level; Table S1: List of the proteins in our dataset; Table S2: Features extracted from the protein dihedral angle curves; Table S3: Properties of the amino acids.
