# Supplementary material for: Improving Protein Subcellular Location Classification by Incorporating Three-Dimensional Structure Information
Source: Biomolecules. 2021 Oct 29;11(11):1607. doi: 10.3390/biom11111607 (PMC8615982; doi:10.3390/biom11111607)
Supplement: Supplementary file 1 [file biomolecules-11-01607-s001.zip › biomolecules-1423122-supplementary.pdf]

SUPPLEMENTARY MATERIALS FOR

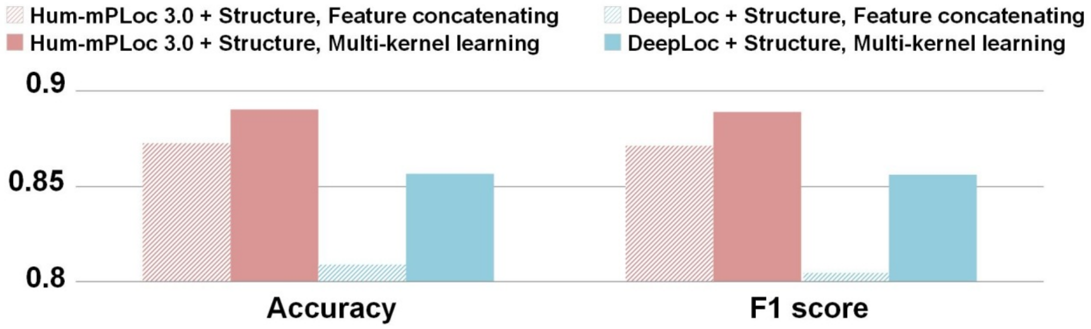

Figure S1. Comparison of the incorporating methods at feature level. Both of the feature concatenating and multi-kernel learning models used the support vector machine classifiers.

Table S1. Features extracted from protein dihedral angle curves.

Features	Description	Dimension
Frequency domain	Maximum amplitude<br>Minimum amplitude<br>Median amplitude<br>Average amplitude<br>Amplitude peak difference<br>Gravity frequency<br>Mean square frequency<br>Root mean square frequency<br>Frequency variance<br>Frequency standard deviation	10
Wavelet transform	high frequency coefficients after 5-layer decomposition of signals	5

Table S2. Properties of amino acids.

Amino acid short form		ALA	ARG	ASN	ASP	CYS	GLN	MET	GLU	GLY	HIS	ILE	LEU	LYS	PHE	PRO	SER	THR	TRP	TYR	VAL
General properties	Charged-W	0	1	0	1	0	0	0	1	0	0	0	0	1	0	0	0	0	0	0	0
	Polar-W	0	0	1	0	1	1	1	0	0	1	0	0	0	0	0	1	1	1	1	0
	Hydrophobic-W	1	0	0	0	0	0	0	0	1	0	1	1	0	1	1	0	0	0	0	1
	Hydrophobic-S	1	0	0	0	0	0	0	0	1	0	1	1	0	1	1	0	0	1	1	1
	Moderate	0	0	0	0	1	0	1	0	0	1	0	0	0	0	0	0	0	0	0	0
	Hydrophilic	0	1	1	1	0	1	0	1	0	0	0	0	1	0	0	1	1	0	0	0
	Polar-S	0	0	1	0	1	1	1	0	0	0	0	0	0	0	0	1	1	0	0	0
	Aromatic	0	0	0	0	0	0	0	0	0	0	0	0	0	1	0	0	0	1	1	0
	Aliphatic	1	0	0	0	0	0	0	0	0	0	1	1	0	0	0	0	0	0	0	1
	Acidic	0	0	0	1	0	0	0	1	0	0	0	0	0	0	0	0	0	0	0	0
	Basic	0	1	0	0	0	0	0	0	0	1	0	0	1	0	0	0	0	0	0	0
	Negative charged	0	0	0	1	0	0	0	1	0	0	0	0	0	0	0	0	0	0	0	0
	Neutral	1	0	1	0	1	1	1	0	1	0	1	1	0	1	1	1	1	1	1	1
	Positive charge	0	1	0	0	0	0	0	0	0	1	0	0	1	0	0	0	0	0	0	0
	NH2-Pka	9.9	9	9	9.6	11	9	9.2	10	9.6	9	10	9.6	10	9	11	9	9	9.4	9	9.7
	COOH-Pka	2.4	2	2	1.9	1.7	2	2.3	2	2.3	2	2	2.4	9	3	2	2	2	2.4	2	2.3
Properties based on hydrophilicity and hydrophobicity	Strongly hydrophilic or polar	0	1	1	1	0	1	0	1	0	1	0	0	1	0	0	0	0	0	0	0
	Strongly hydrophobic	1	0	0	0	0	0	1	0	0	0	1	1	0	1	0	0	0	0	0	1
	Weakly hydrophilic or weakly hydrophobic	0	0	0	0	0	0	1	0	0	0	0	0	0	0	0	1	1	0	1	0
	Proline	0	0	0	0	0	0	0	0	0	0	0	0	0	0	1	0	0	0	0	0
	Glycine	0	0	0	0	0	0	0	0	1	0	0	0	0	0	0	0	0	0	0	0
	Cysteine	0	0	0	0	1	0	0	0	0	0	0	0	0	0	0	0	0	0	0	0

Note: '-W' represents the web reference of proteins and '-S' represents the soluble reference. '1' means the amino acid has corresponding property and '0' means no such property. '-Pka' represents dissociation constants.
